# Trapped Ion Mobility Improves Annotation Accuracy in LC-HRMS Screening Applications for Exposomics

**DOI:** 10.1021/acs.analchem.5c04665

**Published:** 2025-10-31

**Authors:** Carolin Huber, Nadin Ulrich, Martin Krauss

**Affiliations:** † Department of Exposure Science, 28342 Helmholtz Centre for Environmental Research - UFZ, Permoserstr. 15, Leipzig 04318, Germany

## Abstract

Ion mobility techniques coupled to mass spectrometry, such as trapped ion mobility (TIMS), are promoted to separate analytes from coeluting matrix interferences and to resolve isomers based on their corresponding CCS values. Complementary to the retention time (RT) dimension revealed from liquid chromatography, the collision cross section (CCS) serves as a robust and matrix-independent parameter. We evaluated the advantages of TIMS in the screening of human samples, such as urine, serum, breastmilk, and matrices relevant for exposure analysis, such as dust and wastewater. We conducted a screening library for 769 environmental contaminants, which resulted in a total of 948 CCS values (594 positive and 354 negative ionization modes). We screened for the potential co-occurrence of interfering compounds originating from five different matrix backgrounds, leading to peaks with similar *m*/*z* and RT but differences in the mobilograms. For all matrices combined, 112 peaks with different mobility values relative to the reference standard were found. Our evaluation highlights the benefits of TIMS in reducing the number of inconclusive assignments through the separation of coeluting compounds and background noise and gaining a high MS^2^ coverage for low-abundant ions. These advantages are beneficial especially for suspect screening applications, where broader RT windows are necessary.

## Graphical abstract

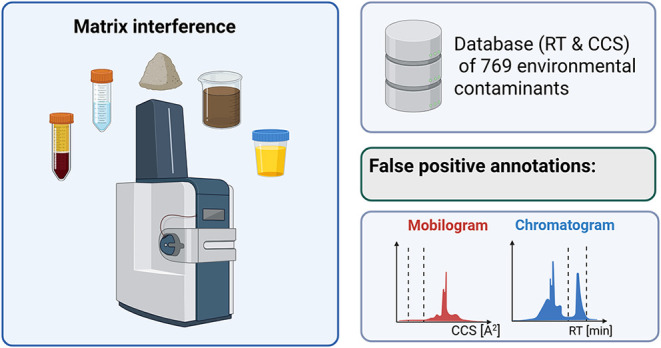

## Introduction

The widespread use of chemicals in everyday life significantly exposes humans and the environment, posing potential risks to human health and ecosystems.^1^ Dedicated target analysis approaches cannot cover only the variety of chemical contaminants and their transformation products. Thus, screening methods based on liquid chromatography coupled with high-resolution mass spectrometry (LC-HRMS) are required. In target screening using high-resolution mass spectrometry (HRMS), high numbers of preselected compounds are analyzed simultaneously, requiring in-house availability of reference standards. Suspect screening allows for a broadening of the scope of analysis by addressing compounds without reference standards, but relying on their expected mass-to-charge ratio (*m*/*z*), isotope pattern, and other parameters that are received from another laboratory or predicted.^2^ In both target and suspect screening, false positives at MS^1^ level occur frequently in complex environmental and biological matrices due to coeluting isomeric or isobaric compounds, which form interfering ions, adducts, or in-source fragments.^3^ In addition to that, interferences that mask compounds of interest through high background levels may result in false negatives, especially when only trace levels are present. Due to an insufficient separation in the LC dimension, MS^2^ spectra may contain fragments from other compounds or stereoisomers within the precursor isolation window, leading to decreased spectral matching values^4^ or difficulties in isotope pattern evaluations, which are often needed for molecular formula annotations.^5^

In recent years, ion mobility separation (IMS) and, later, trapped ion mobility separation (TIMS) have been introduced as an additional separation dimension to HRMS.^6^ While its first applications started in proteomics,^7^ it is increasingly used in recent years in small molecules analysis, e.g., in lipidomics^8,9^ and metabolomics^10^ and has begun to reach the field of exposomics and environment,^11–13^ for example, for PFAS analysis.^14–16^ In TIMS, ions enter the analyzer with an electrical field by drag of a gas flow, where a gradual lowering of the electrical force leads to the elution of ions according to their mobility in the gas phase.^17^ The collisional cross section (CCS) of the ion can be calculated on the basis of its electrical field (1/K_0_) of elution. Although CCS values cannot be directly calculated in TIMS without calibration through established reference compounds with known CCS values,^18^ comparable and highly reproducible CCS values are reported.^19,20^ The advantages of TIMS involve a high-resolution IMS (resolving power 200–400) combined with avoiding a loss in sensitivity through the generation of two trapping regions in the ion mobility cell, which results in simultaneous trapping and analysis of ions. However, this only applies if the same values are used for the accumulation and ramp times. Then, the benefit of high-duty cycles can be achieved with only minimal loss of sensitivity.^21^ Further implementations promote the improvement of the MS^2^ coverage by using parallel accumulation-serial fragmentation (PASEF) through synchronization of the TIMS cell with a fast quadrupole analyzer.^7^

For exposomics and other small-molecule science applications, the overall benefits of IMS rely on the assumption that (1) the analytes of interest, as well as the sample matrix, show a sufficient diversity in CCS values and (2) sufficient separation can be achieved by current-state IMS technology. Therefore, we evaluated a set of more than 769 chemicals of interest in the field of exposomics, if an additional ion mobility separation allowed for a potential avoidance of interfering ions in different sample matrices. Additionally, we also assessed the performance of the method performance with regard to MS^2^ spectra quality and MS^2^ coverage and evaluated the influence of different TIMS parameters.

## Materials and Methods

### Sample Preparation

We used pooled urine, sheep serum, breastmilk, house dust, and wastewater treatment plant (WWTP) influent, as often used in the context of wastewater-based epidemiology, as exemplary sample matrices to evaluate the performance of the TIMS separation. A detailed description of the sample preparation is given in Table S1. All samples were spiked before analysis with the same internal standard mixture of 40 labeled compounds (see Table SX2). After sample extraction, aliquots of each sample matrix were spiked to reveal replicates with two different concentration levels (10 and 100 ng/mL concentration in the sample extract) of a reference mixture containing compounds relevant for exposomic analysis (see Table SX1 for a list of all compounds). Higher levels were used for dust samples, where 50 and 500 ng/mL were spiked, as higher matrix effects and analyte concentrations were expected. At the beginning and end of the measurement sequence, the sole reference mixtures (concentrations 0.5, 1, 5, 10, 500 ng/mL) were measured in addition to the solvent blanks spread over the sequence.

### LC-TIMS-HRMS Measurements

Samples were measured with reversed phase liquid chromatography (for more details, see Table S2) coupled to a TimsTof Pro 2 (Bruker, Bremen, Germany) in positive and negative ionization mode using a VIP-HESI source (source parameters see Table S3). Further applications of instrument and calibration parameters are summarized in Table S4. The inverse reduced mobility (1/*K*_0_) values were measured with trapped ion mobility in a range of 0.45–1.45 V*s/cm^2^ with a ramp and accumulation time of 100 ms and nitrogen as carrier gas. The instrument set the TIMS cartridge tunnel pressure to 2.64 mbar/0.768 mbar (in/out). The ion charge control (ICC) was activated to reduce the TIMS tunnel saturation and the effects of the space charge effects, with a limit of 7.5 × 10^6^ for the number of charges in the TIMS cell.

For calibration, 20 μL of a 1:1 mixture (v/v) of Agilent ESI-Low Concentration Tune Mix and sodium formate buffer solution (10 mM) was injected through a 6-port valve with a sample loop injected before each analysis. The ion mobility using nitrogen gas was recalibrated using the masses of the Agilent tune mix (*N* = 6) and the values of the “CCS compendium” comprising the CCS values calculated by Stow et al.,^16^ while the mass accuracy was calibrated using the calculated masses of the sodium formate clusters (*N* = 12). Data-dependent MS^2^ spectra (dd-MS^2^ were acquired with PASEF^7^ using a cycle time of 0.53 s for two MS^2^ ramps. The isolation window was set to 1 *m*/*z*, employing a combined collision energy of 20 and 50 eV. The selection of precursors was carried out with an active exclusion for 0.1 min. All samples in spiked and native form were injected as duplicates, measured with TIMS separation and additionally with switched-off ion mobility (detailed parameters, see Table S5). In addition, a replica of urine samples (spiked and native) was also measured with three different ramp times (100, 200, 300 ms).

Raw data (as Bruker *.d files) have been deposited to the EMBL-EBI MetaboLights database^22^ with the identifier MTBLS11753 and are accessible directly at https://www.ebi.ac.uk/metabolights/MTBLS11753.

### Data Processing

The manual evaluation and extraction of the target compounds was performed in TASQ version 2024b (Bruker, Bremen, Germany), with all parameters for the extraction and annotation of the analytes summarized in Table S6. Data processing steps included automatic mass and ion mobility recalibration, generation of extracted ion chromatograms (EICs), extracted ion mobilograms (EIMs), and CCS calculation based on the measured 1/*K*_0_ values. To achieve an automated open source extraction of EIC and EIM, the Python package AlphaTims^23^ was used. The Python script for data evaluation is available on GitHub (https://github.com/chufz/timstofscreener). An additional evaluation of the raw data from the different sample matrices was performed using the raw data overview and heatmap graphs in MZmine (version 4.1.0).^24^

True annotations were defined within a *m*/*z* window of 5 ppm, ΔRT of 0.25 min, and a ΔCCS of less than 3%. To differentiate any additional peak that occurs in the EIM as interference, we define a ΔCCS of >5% as boundary.

Metaboscape version 2024b (Bruker) was used to generate a feature list that included an ion deconvolution step of all native matrices and to perform a spectral library search. The applied workflow and parameters are summarized in Table S7. For annotation of the spiked compounds, the tandem mass spectral library of NIST 2020 and MassBank EU (version 06–24) were used. All features that showed a higher mean abundance in the blank sample were removed. The overall measurement performance was evaluated using the Bruker RealTimeQC module (see Figures S1 and S2). Further structure elucidation of the interferences was performed using MetFrag^25^ and CCSBase^26^ to predict the CCS values of the candidates.

## Results and Discussion

### CCS Database Generation for Reference Analytes

A screening database, comprising CCS and RT values, was generated for the analytes of the reference standard mixture and used for a screening workflow on the spiked and nonspiked matrices. In total, we could detect 769 unique environmental contaminants, for which 594 single charged ions were annotated in ESI+ and 354 single charged ions in ESI- (see Table SX1). The reported values are averaged over five injections of the reference mixture at different concentrations. Some small overlap of compounds with previously reported CCS values were found. We achieved a mean difference of 0.63% to Belova et al.^12^ (*N* = 39) and 0.99% for Celma et al.^13^ (*N* = 126), see Table SX6. The intrabatch stability of CCS was assessed over the spiked internal standards (see Figures S1,S2 and Table SX2). The mean of all internal standards for the measured ΔCCS was 0.33% for the solvent mixtures and 0.31% for all sample matrices. This indicates that there are no influences introduced by the sample matrix on the TIMS stability.

For 94 chromatographic peaks of the analytes in ESI+ and 60 in ESI- mode, more than one peak was observed in the EIM, and the extracted dd-MS^2^-PASEF spectra for the integrated mobility range revealed a match to the analyte for all peaks (see Table SX1 and Figure S3 for all additional CCS values). The same pattern of peaks was also observed in all solvent mixtures and in the spiked matrices. Several reasons explain the assignment of several peaks in the EIM to one analyte, such as coeluting isomers of the substance, but also different ionization sites (protomers), solvent-analyte clusters,^27^ or dimerization effects. For further analysis of the spiked matrices, only one peak was determined as a characteristic peak for each compound and added to the database to simplify the annotation procedure. However, additional CCS values are also reported separately. The selection was based on the lowest CCS value and if an R^2^ > 0.8 for the signal response over the solvent calibration was observed. For the evaluation of interfering ions, any additional CCS values identified were disregarded. However, we want to note that the selected EIM signals with the lowest 1/K_0_ were not always associated with a higher abundance than the disregarded peaks, especially for the measurements of the highest reference mixture concentration.

Dimerization was previously postulated for fluorinated compounds.^28,29^ The formation of single-charged dimers from the VIP-HESI source may lead to a different necessary trapping time in the TIMS cell, but separation into monomers or other rearrangements appears before the analyzer, which leads to signals with the same *m*/*z* as that of a monomer. We evaluated the signal abundance of the peaks in the EIM in correlation with the concentration (see Figure S4 for one example), as any formation of dimers during the ionization should be concentration dependent. We compared the signal responses of both peaks to hypothesize the potential of the monomer (lower CCS) and dimer (higher CCS) as a function of the concentration.

All final CCS values (assigned to the potential monomer) are displayed in correlation with their *m*/*z* value in Figure 1 for each ionization mode. As previously reported,^30^ most compounds are located near a trendline, as CCS in general increases with molecular mass. A lower CCS for a given *m*/*z* can be observed for [M-H]^−^ ions of polyhalogenated compounds, among them mainly per- and polyfluorinated acids, which have already been reported as a promising approach for nontarget screening applications for unknown PFAS.^16^ Furthermore, lower CCS to *m*/*z* ratios are found for iodine-containing X-ray contrast agents with high molecular masses, such as diatrizoate, iohexol, iopromide, iopamidol, and iomeprol, but also brominated compounds, such as hexabromo-cyclododecane, tetrabromobisphenol A, and 2,4,6-tribromophenol. Even compounds with a lower carbon/halogen ratio, such as fipronil and its transformation products (desulfinyl, sulfone, and sulfide), tritosulfuron, and sucralose, are observed below the general trendline.

**Figure 1:**
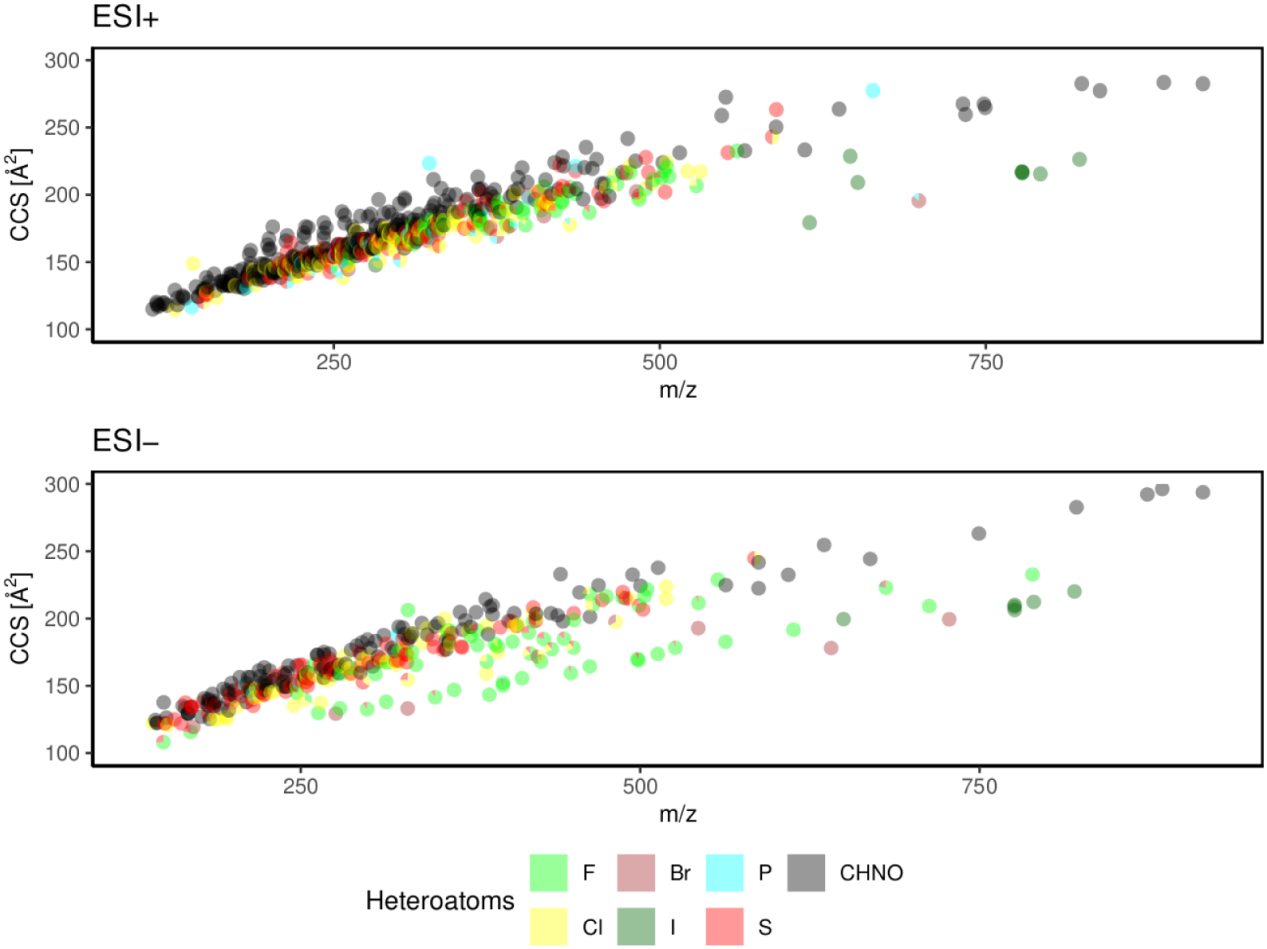
Distribution of the CCS vs *m*/*z* plot for the database of CCS values for 769 compounds, including 594 ions in ESI+ and 354 ions in ESI-. If the compound contains one of the heteroatoms F, Br, P, Cl, I, or S, the point is marked by that color. Compounds that contain only C, H, N, and O are kept in black.

Accurate discrimination between highly similar isomers, such as those differing only by the position of a single functional group, is critical for ion mobility separation. If a separation is achieved, it will depend on (i) the resolving power of the ion mobility (IM) spectrometer and (ii) the reproducibility of collision cross section (CCS) values within the analytical matrix. Some examples observed in the reference mixture are simazine (CCS = 142.98 Å^2^, RT = 11.98 min) and desethyl-tebuthylazine (CCS = 144.23 Å^2^, RT = 11.98 min), or isomers such as 1,7-diaminophenazine (CCS = 143.60 Å^2^, RT = 8.40 min) and 2,8-diaminophenazine (CCS = 143.53 Å^2^, RT = 8.22 min), where a separation in the IMS was not possible. For a separation of these analytes of interest from potential interferences originating from the sample matrices, we assume enough structural differences that a sufficient separation should be achieved.

The number of analytes detected in the solvent was compared for three different concentrations between TIMS switched on and off (see Figure S5). For high concentrations (500 ng/mL), no significant differences were observed. For lower concentrations, we observed differences between the ion modes. While in positive mode, a higher number of analytes could be detected with TIMS separation, a lower number could be detected in negative mode. Several factors impact the sensitivity, such as a decreased signal-to-noise ratio when the TIMS separation is used, as interfering ions are separated. A loss of sensitivity can be avoided by a full-duty cycle by equal accumulation and ramp times. Although this might be the ideal case, applying an ion charge control with complex matrices, the actual accumulation times during the measurement might be lower (see Figure S6 as an example for WWTP influent). For these cases, the instrument will set a lower accumulation time than ramp time, leading to a difference in sensitivity for the TIMS measurements.

### Sample Matrix Characterization

For further evaluation, we assume that the complexity of the matrix increases with an increasing number of detected nontarget features in each sample, resulting in an order of breastmilk < serum < dust < urine < WWTP influent (see Figure 2). When plotting the nontarget feature list containing all types of ions for the different sample matrices as CCS against the *m*/*z* values, we observe three clearly distinguishable feature groups along trendlines. As previously reported,^30^ these are mainly related to three different charge states, with higher CCS values for double-charged (“middle” CCS range of 300–500 Å^2^) and for triple-charged ions ("upper" CCS range 500–700 Å^2^), which particularly occur in positive ionization mode. Wastewater, for example, contains a large number of polyethylene glycols, which can form multiple charge states in ESI+ through their large number of ether bonds, forming homologue series spaced by 44 Da. Multiple charged ions found for breast milk are potentially represented by the large number of oligosaccharides. Furthermore, the feature list is also affected by the selected sample preparation method, such as lipid removal for breastmilk, protein precipitation for serum, liquid extraction for dust, and solid phase extraction for urine and wastewater influent. When aligning the features between the matrices, the highest number of similar features was found between the wastewater influent and urine (*N* = 2,679 for both modes of ions), followed by the WWTP influent and dust (*N* = 2,061, see Venn diagram in Figure S7).

**Figure 2:**
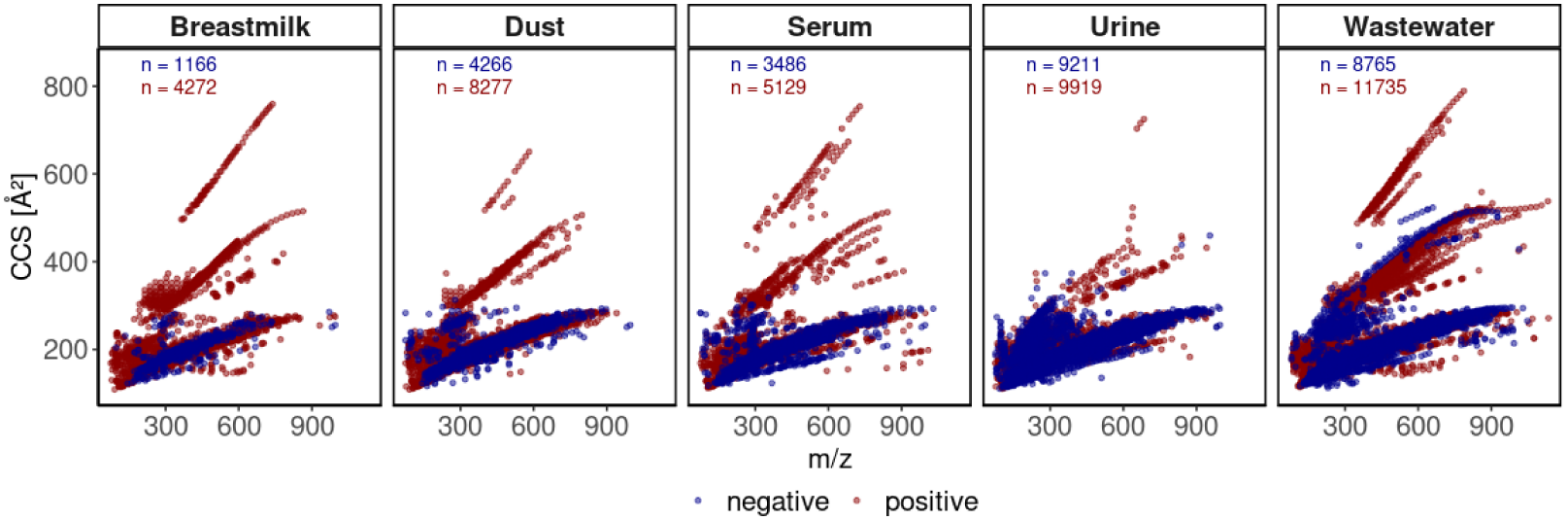
CCS vs *m*/*z* plot of the feature table for different native sample matrices after blank correction, measured in negative (blue) and positive (red) ionization modes, including all different charge states. The number of features (*n*) is given for both ionization modes.

### Evaluation of Potential False-Positive Annotations in Screening Applications

The overall separation performance between the analytes from the matrix background is achieved through a high orthogonality between the separation in the dimensions of chromatographic and ion mobility^31^ (see Figure S8). The reverse phase LC method used in this study is quite typical for screening applications^32^ (C18 column and acidic eluent in ESI+/basic eluent in ESI-) and was therefore used to evaluate the additional benefit of the TIMS dimension on the overall separation efficiency.

We evaluated the number of annotations based on the *m*/*z* of the expected ion (window of 5 ppm) and RT (window of ±0.25 min) in the EIC, where a different peak is present in the extracted mobilogram (EIM), but with a different MS^2^ spectra. The five different native sample matrices were taken as exemplary sample backgrounds (Figure 2) for all analytes (see Figure 1). The EIM of the integrated chromatographic peak of all ions of the analytes (*N* = 948) was compared with the EIM of the pure solvent reference standards measurements for additional peaks that appear only in the sample matrices. To be conservative for potential shifts, we applied a CCS threshold of >5% to associate the EIM peak as a distinct peak from the values obtained in the solvent standard. Figure 3 (A+B) shows two prototypic examples appearing, either as coeluting interference or false positive annotation, depending on whether the analyte is also abundant in the sample (see also Table SX3). The absolute numbers underlying the bar plots are given in Table S8.

**Figure 3:**
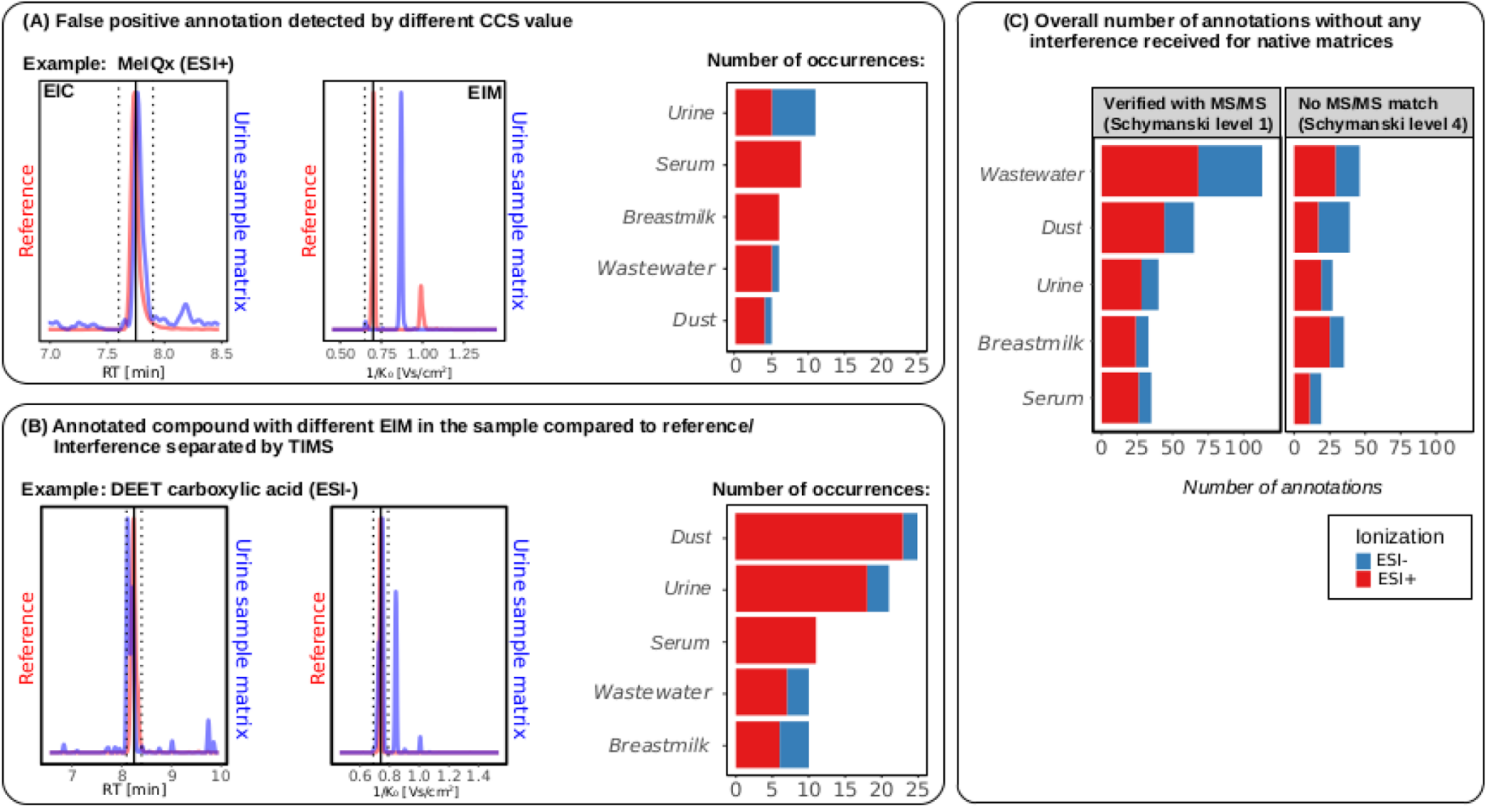
(A) Examples and numbers of possible analytes as false positive annotations through the sample matrix that were separated by TIMS. EIM and EIC of the pooled urine matrix (blue) in comparison to the measured reference standard (red); (B) example of an analyte with an interference that could be separated by TIMS and the numbers of occurrences in each native matrix; (C) bar graphs summarizing the number of true annotations revealed without any observed interference for comparison.

In the absence of the true analyte, coeluting interference would result in a false positive annotation without ion mobility. An example of this case is the detection of the mutagenic and cancerogenic compound 2-amino-3,8-dimethylimidazo­[4,5-*f*]­quinoxalin (MeIQx) in the pooled urine sample (see the example in Figure 3A). The EIC for the native urine sample and the reference standard only shows a small ΔRT of 0.04 min, while the EIM shows a different peak with a calculated CCS difference of 29%. When comparing the MS^2^ spectra, no spectral similarity was observed, leading to the conclusion that the peak in the EIC is resolved from a different compound (see Figure S9).

With the presence of the analyte in the sample and no separation through IMS, interference might alter the corresponding isotope pattern and MS^2^ spectrum, leading to a false negative or inconclusive annotation. An example of this case is the detection of the carboxylic acid metabolite diethyltoluamide (DEET) in the pooled urine sample (see Figure 3B). The EIC for the native urine sample and the reference standard shows a ΔRT of 0.13 min, while the EIM shows next to a matching peak a different peak with a calculated CCS difference of 14%. When comparing the MS^2^ spectra extracted for different mobility ranges, no spectral similarity was observed, leading to the conclusion that two different compounds are coeluting in the chromatography.

For both cases (Figure 3A,B), the annotation performance in the positive ion mode is more affected by the matrix interference than in the negative ion mode. For all matrices combined, a total of 112 peaks with different mobility values to the reference standard were found, with 36 peaks assigned as false positive annotations and 76 cases of interference to an existing true annotation of an analyte.

For the detection of 4-(4-hydroxyphenyl)-butan-2-one in urine, a small RT shift (ΔRT = 0.15 min) allowed us to identify the separated interference of a structural isomer by spectral library matching. Here, more evidence is given in the EIM, with a calculated CCS deviation of 32% (see Figure S10A). The detection of 4-hydroxyquinoline in urine exemplifies cases where several potential structural isomers might not be clearly identified by MS^2^, as all possess a low fragmentation efficiency at the collision energies used and likely rather similar fragmentation spectra. In these cases, only minor changes in EIC (ΔRT = −0.12 min) can raise suspicion, but the additional peak in the EIM (ΔCCS = 14%) provides evidence that another structural isomer is also present (see Figure S10B). In the majority cases, however, structure elucidation of the interference remains difficult, as not only one precise underlying chemical composition can be identified but rather a high background noise. One of these cases represents the annotation of cotinine in sheep serum (see Figure S10C). This is especially the case for compounds exhibiting a poor chromatographic peak shape.

In addition, Figure 3C summarizes the general annotations (*N* = 472) achieved for the unspiked matrices (see Table SX4), for which a mean *m*/*z* error of 1.04 ppm, an RT error of 0.03 min and a CCS deviation of 0.6% was observed. The high number of Schymanski level 1 annotations (confirmed by MS^2^ spectra)^33^ versus level 4 annotations (no MS^2^ coverage or poor matching due to low intensity), which can be observed in Figure 3C for native samples, highlights the benefits of high spectral coverage of this acquisition method (see further evaluations in Evaluation of dd-MS2-PASEF Spectra Quality and Coverage). The number of false positives (Figure 3A) identified by TIMS did not exceed more than ten for each matrix, which accounts for ∼ 1% of the total number of evaluated ions. For most interferences, poor isotope pattern matching and a high *m*/*z* or RT deviation close to the maximum acceptable limit (RT ±0.25 min and *m*/*z* error of ±5 ppm) also provided some insights that the annotation might not be accurate.

For 30% of the additional peaks in the EIM, a ppm value >2.5 ppm was observed. Therefore, a high fraction of interferences separated by TIMS might be isobaric compounds or background noise rather than structural isomers. The number of annotations that show a sufficient separation of interference with TIMS (Figure 3B) represents 14% of the confirmed annotations achieved (Schymanski level 1) in all matrices, with the highest fraction observed for urine (54%). This suggests that the benefits of TIMS are more on the side of avoiding false negatives or inconclusive assignments than on actual false positives when taking into account a workflow that includes MS^2^ as follow-up step for confirmation.

The evaluation described above was conducted on a set of target compounds, but a potential suspect screening workflow was assessed. With regard to suspect screening applications, the applied values for will, however, rely on predicted CCS and RT values or databases generated from measurements by different instruments. We compared the predicted CCS values with our experimental values for a prediction model, CCSBase.^26^ A comparison of predicted CCS_[M+H]+_ with our experimental values showed an R^2^ of 0.95, with an RMSE of 7.27 Å^2^ (equals 4.2%, see also Figure S11).

Compared to the mean deviation observed for the internal standards (max. error of approximately 3% for all labeled compounds throughout the sequence), we conclude that for suspect detection with predicted CCS values, annotation windows of <5% can be applied. The observed interferences mentioned above appeared with a mean deviation larger than ±15 Å^2^ to the analytes’ CCS values (see Table SX5), concluding that these applied CCS annotation windows will be sufficient for the separation of interferences and barely influence the annotation performance. However, we want to mention that this only accounts for a separation of xenobiotic analytes from interfering compounds originating from the sample matrix, which might show a considerable structural difference. Any separation between isomers with closely related structures might be more limited. Differences in the CCS values of structurally similar compounds are assumed to be smaller than the prediction accuracy of the model; therefore, a predicted CCS will not help in compound annotation.

This good prediction performance for CCS values stands in contrast to RT predictions, where highly accurate prediction models are still lacking.^34^ A prediction model based on the reference standards measured in positive ion mode generated with RTPred^35^ showed a prediction performance with an R^2^ of 0.65 for the data of this study, with an MAE of 1.71 min (see also Figure S12). This deficit results in the need for a broader annotation window in suspect screening applications, and therefore in a higher probability of false positive annotations. We reevaluated the data with a broader RT window by a factor of 10. This increase in the RT window from ΔRT= ±0.25 min to ΔRT= ±2.5 min introduced an average of 16 false positive annotations in each sample matrix (2% of the total number of analyte ions), with the highest number observed for dust extraction (*N* = 27). A list of false-positive annotations introduced through the larger RT window is summarized in Table SX5.

### Evaluation of dd-MS^2^-PASEF Spectra Quality and Coverage

Achieving a high dd-MS^2^ coverage during the measurement is critical for screening applications, as structure elucidation or confirmation relies on the presence of MS^2^ information, alongside with a high spectral quality. Several publications in proteomics and lipidomics have already reported and evaluated the high advantage of dd-MS^2^-PASEF accumulation in increasing spectral quality and coverage.^36,37^ As shown in Figure 4, the influence of the sample matrix on spectral coverage increases with matrix complexity, resulting in lower spectral coverage. Additionally, the spectral quality decreases, leading to decreased spectral matching scores, especially for low-level spiked samples. We observed a spectral library search annotation coverage of 64–93% (100 ng/mL) and 32–84% (10 ng/mL) in the spiked matrices compared to the solvent mixture at the same concentration.

**Figure 4:**
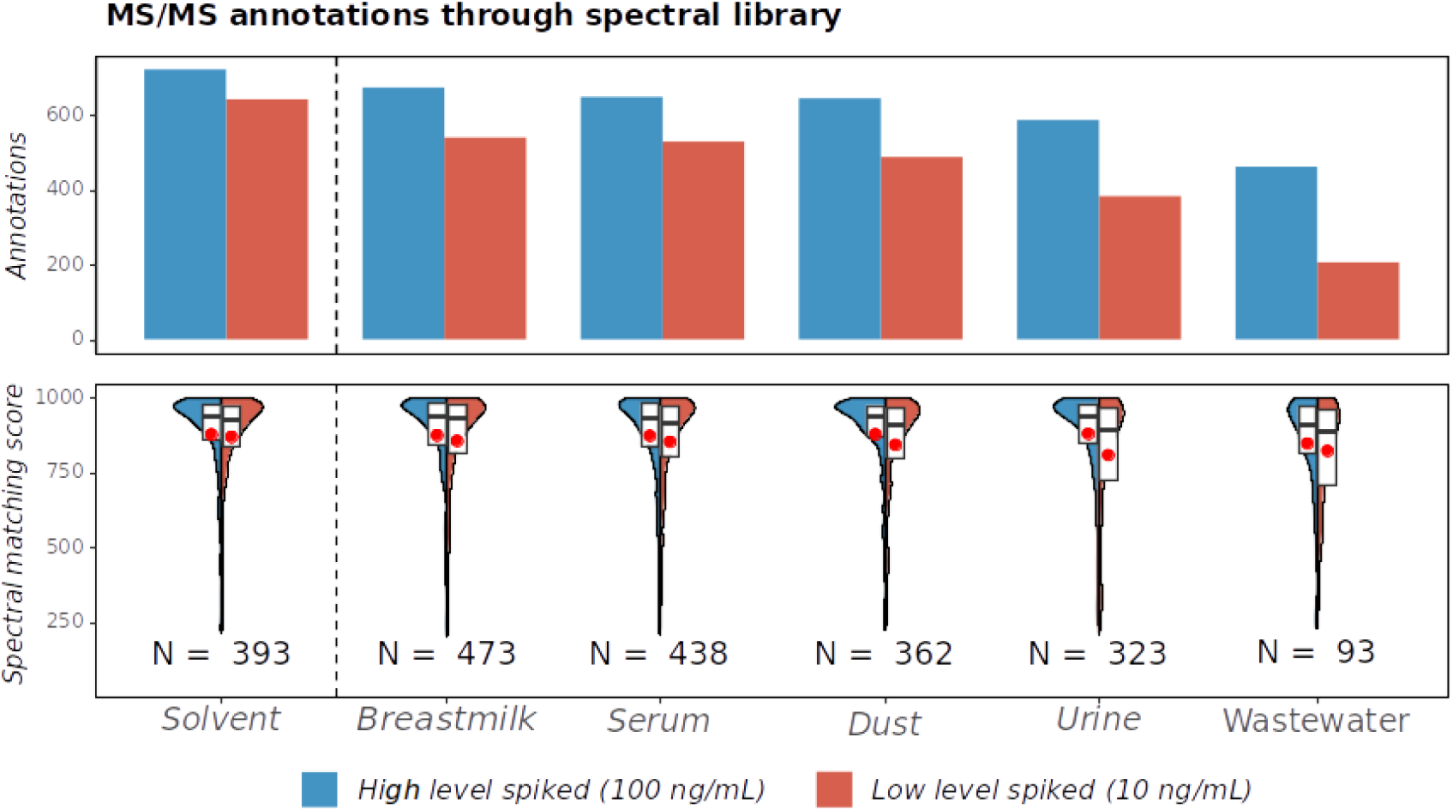
Dependency of the MS^2^ spectral coverage (top) and distribution of the spectral matching score against the NIST and MassBank library (bottom) on the analyte concentration and background matrix. The results include both ionization modes. The distributions of spectral matching scores of all annotations found for both spiking levels are displayed as violin plots with annotated mean (black) and median (red).

We evaluated the spectral quality based on the matching scores achieved for a spectral library matching. The violin plots in Figure 4 compare the distribution of scores obtained for the spectral matching at two different spiking levels. For comparability of the matching scores achieved against the spectral library, only the overlap of annotations achieved in both spiking levels are shown. Generally, lower scores are observed for complex matrices like wastewater and urine, with a further decrease in the mean match scores achieved for lower spiking levels, which can be explained by interfering ions in the precursor window, leading to decreased spectral quality and lower scores. Comparison of spectral quality to switched-off TIMS measurements remains difficult, as several other parameters influencing the results, such as the applied precursor target intensity, which cannot be easily kept comparable among both acquisition modes. However, for two analytes with observed interferences in the urine matrix, which were only poorly separated with a ramp time of 100 ms, we could achieve an increase in spectral quality through better separation with higher TIMS resolution by increasing the ramp time to 300 ms (see Figures S13 and S14).

## Conclusions

The annotation of suspects or targets in HRMS^1^ data for complex biological sample matrices requires caution, as the analytes of interest are merely a needle in the haystack of the sample matrix. The number of chromatographic interferences that were separated through TIMS (*N* = 112) for all observed analytes (*N* = 769) in five matrices indicates the probability of how often false positive MS^1^ tentative annotations will occur in exposomic screening applications, if they would only rely on an MS^1^ annotation with *m*/*z* and RT but no further MS^2^ confirmation. Our exemplary study demonstrated the benefits of ion mobility separation for the annotation and structural identification of contaminants in complex matrices.

Especially when the applied screening workflow relies on predicted RT values, broader annotation windows are necessary to account for prediction inaccuracies, further increasing the number of inaccurate tentative annotations. When additionally CCS windows are applied for annotation, a higher specificity is achieved by separating isomeric and isobaric compounds that fall into the same RT window as the intended suspects.

However, using TIMS and predicted CCS values does not replace sufficient upstream chromatographic separation, as well as all other classical identification criteria, such as MS^2^ spectra comparison and isotope pattern analysis. However, the applicability of these identification criteria is limited. Especially due to the requirement for a sufficient abundance, which is not always achievable, especially when analyzing low-level contaminants. Therefore, robust and effective screening applications should combine all evidence for an annotation provided by mass spectrometry. Here, ion mobility can serve as one contributing factor for revealing high-confidence annotations and improving the overall annotation performance. To obtain a large number of samples and suspects, these confirmation steps should be automatically evaluated and integrated in a user-friendly manner.

To conclude, our observations highlight the benefits for exposomics of high-resolution IMS separation in combination with a high MS^2^ spectra coverage. However, other ion mobility techniques, such as traveling wave ion mobility (TWIMS) when applied with Structures for Lossless Ion Manipulations (SLIM) or cyclic ion mobility (cIMS), have also shown high resolution ion mobility.

## Supplementary Material





## Notes

Analytical method information, quality control, Tables S1–6, comparison of primary and additional CCS values, concentration dependence of different extracted ion mobilograms, Venn diagram between different matrices, comparison between predicted and measured CCS values, Figures S1–S14 (PDF)
Tables of all reference analytes with CCS values (SX1), spiked internal standards (SX2), observed additional peaks in extracted ion mobilograms (SX3), annotated compounds (SX4), false positives occurring as results of a wider annotation range as a suspect screening scenario (SX5) and comparison with literature values (SX6)­(XLSX)

## ‡

Department of Environmental Chemistry, Eawag - Swiss Federal Institute of Aquatic Science and Technology, Ueberlandstrasse 133, Duebendorf 8600, Switzerland
