# Supplementary material for: Trapped Ion Mobility Improves Annotation Accuracy in LC-HRMS Screening Applications for Exposomics
Source: Anal Chem. 2025 Oct 31;97(44):24608–15. doi: 10.1021/acs.analchem.5c04665 (PMC12613149; doi:10.1021/acs.analchem.5c04665)
Supplement: Supplementary file 1 [file ac5c04665_si_001.pdf]

Trapped ion mobility improves annotation accuracy in LC-HRMS screening applications for exposomics

Carolin Huber[1,2*], Nadin Ulrich[1,] Martin Krauss[1]

[1]Department of Exposure Science, Helmholtz Centre for Environmental Research - UFZ Permoserstr. 15, 04318 Leipzig, Germany
[2]current address: Department of Environmental Chemistry, Eawag - Swiss Federal Institute of Aquatic Science and Technology, Ueberlandstrasse 133, 8600 Duebendorf, Switzerland

Supporting Information

Table of Content

Tables

Table S1: Detailed sample preparation procedure

Table S2: Chromatic separation details

Table S3: VIP-HESI parameters

Table S4: Timstof Pro 2 settings

Table S5: Timstof Pro 2 settings (for mobility switched off measurments)

Table S6: Metaboscape data processing settings

Table S7: TASQ data processing settings

Table S8: Overview of the numbers of achieved annotations

Figures

Figure S1: Measurement stability in positive ionization mode

Figure S2: Measurement stability in negative ionization mode

Figure S3: CCS over m/z for all peaks related to the spiked compounds

Figure S4: Example of the concentration dependency of the EIM

Figure S5: Comparison of the number of annotations between TimsON and TimsOFF

Figure S6: Influence of the accumulation time on the signal intensity over the measurment

Figure S7: Venn diagramm showing the overlap of similar features between the matrices

Figure S8: Retention time over m/z for all sample matrices

Figure S9: Example analyte showing a difference in ion mobilogram and MS2 spectra

Figure S10: Selected analytes with interferences, showing EIM and EIC

Figure S11: Experimental vs predicted CCS values

Figure S12: Experimental vs predicted RT values

Figure S13: Influence of the TIMS resolution on spectral quality for the analyte desloratadine in urine

Figure S14: Influence of the TIMS resolution on spectral quality for the analyte raloxifene in urine

Table S1: Sample preparation procedure for five exemplary sample matrices.

Pooled urine:	A pooled urine sample (N=25) was sampled among the laboratory participants and was prepared using a similar sample protocol as in a previous large-scale cohort study [21, 22]. In brief, 500 µL of pooled urine were diluted in 500 µL of a phosphate buffer solution (pH 6.8) and loaded on HLB solid phase cartridges (30 mg, 30 µm particle seize, CHROMABOND® Machery Nagel, preconditioned with 500 µL of MeOH and equilibrated with 500 µL of H_2O), washed with 200 µL of H_2O and extracted with 1 mL of ACN:H_2O (90:10 v/v). The eluate was evaporated at room temperature using 10 µL of DMSO as a keeper and reconstituted in 100 µL of H_2O and 5 µL of IS mixture (see Table SX2) in MeOH (10 µg/mL).
Sheep serum:	Protein precipitation was performed as simple sample preparation step [23]. In brief, 200 µL of serum was combined with 800 µL of ACN in a filtration vial (GVS Filter Technology, PFTE membrane, 0.2 µm) and left for 1 h at -20°C prior to filtration. The filtrate was evaporated under a gentle nitrogen stream (heated at 40°C) until near dryness and reconstituted with 100 µL of H_2O/ACN 90:10 (v/v) and 5 µL of IS mixture (see Table SX2) in MeOH (10 µg/mL).
Breastmilk sample:	Sample preparation was based on a previous publication for suspect screening of halogenated compounds in breast milk [24]. In brief, an aliquot of 400 µL of breastmilk was added to 1.6 mL of ACN, vortexed for 1 min, and centrifuged for 10 min at 3500 rpm. The supernatant was loaded on Captiva EMR-Lipid cartridges (60 mg, Agilent, preconditioned with 10 mL of 80:20 ACN/H_2O v/v) and eluted. The eluate was concentrated under a gentle nitrogen stream (heated at 35°C) until near dryness and reconstituted with 50 µL of MeOH/H_2O 70:30 (v/v). A volume of 2 µL of IS mixture (10 µg/mL) in MeOH (see Table SX2) was added.
Wastewater influent:	A 24-h composite sample of an exemplary influent of a wastewater treatment plant was extracted based on an in-house method. In brief, after filtration through Whatmann GF/F microfiber filter (0.55 µm, 135 mm diameter), 650 mL of H_2O was solid-phase extracted using Chromabond HR-X cartridges (85 µm, 200 mg, Macherey-Nagel, preconditioned with 5 mL of each ethyl acetate and methanol, and 10 mL of H_2O). After elution using 5 mL of ethyl acetate, followed by 5 mL of MeOH, 5 mL of MeOH containing 1% formic acid, and 5 mL of MeOH containing 2% 7 N ammonia in MeOH, the eluent was evaporated to dryness and reconstituted in 650 µL of MeOH. Before analysis, 66 µL of MeOH, 30µL of LC-MS grade H_2O, and 5 µL of an IS mixture (see Table SX2) in MeOH (10 µg/mL) were added to 1 µL of the reconstituted sample.
Dust sample:	One exemplary sample was prepared based on a previous publication for suspect screening in house dust [25]. In brief, 20 mg of dust (<100 µm particle size) were extracted twice with 2.5 mL of *n*-hexane/acetone (v/v) in an ultrasonic bath for 5 min. The supernatant was evaporated under a gentle nitrogen stream (heated at 35°C) until near dryness and reconstituted in 140 µL of MeOH and 30 µL of H_2O, and 50 µL of IS mixture (1 µg/mL) (see Table SX2).

Table S2: Details on the chromatographic method and sample injection.

**Injection volume:**	5 µL for all measurements
Vendor of LC pump, oven, and autosampler:	UltiMate LPG-3400 quaternary pump<br>WPS-3000 autosampler<br>TCC-3000 SD column oven<br>(Thermo Scientific)
Column parameters:	2.1 mm × 100 mm, 1.7 µm particle size, Waters Acquity BEH C18) at 50°C and a flow rate of 0.3 mL/min
Positive mode	Gradient elution with H_2O/MeOH (both containing 0.1% of formic acid and 2 mM ammonium formate) was used with 100:0 at 0 min, 0:100 at 15 min, 0:100 at 21 min, 100:0 at 22 min, and 100:0 at 30 min (v/v)
Negative mode	Gradient elution of water and water/methanol (90:10 v/v), both containing 10 mM ammonium bicarbonate, was used with the same gradient program as employed in positive mode.

Table S3: Ion source parameters applied to the VIP-HESI source.

**Parameter**	**ESI+**	**ESI-**
End plate offset [V]	500	500
Capillary [V]	4500	4500
Nebulizer gas pressure [bar]	2.5	2
Dry gas [L/min]	7	8
Dry gas temperature [°C]	200	230
Sheath gas temperature [°C]	350	400
Sheath gas flow [L/min]	5	4

Table S4: Parameters applied on the TimsTof Pro 2 with ion mobility switched on (TimsON) as a generic screening method. Parameters highlighted with (*) were changed in additional measurements to evaluate their influence on selected measurements as stated in the text.

Parameter	Positive mode method	Negative mode method
Scan range (m/z)	75-1250	
Mobility range (1/K0) [Vs/cm²]	0.45-1.45	
TIMS cardridge tunnel pressure	2.64 mbar/0.768 mbar (in/out)	
Ramp time/ Accumulation time [ms]	100.0 (* additional measurements using 200 and 300 ms)	
Δt1 [V]	-20	20
Δt2 [V]	-120	120
Δt3 [V]	50	-50
Δt4 [V]	50	-50
Δt5 [V]	0	0
Δt6 [V]	20	-20
Funnel 1RF [Vpp]	250	300
Funnel 2 RF [Vpp]	200	200
Multipole RF [Vpp]	200	200
Collision RF [Vpp]	450	450
Quadrupole [eV]	5	5
TOF transfer time [µs]	65	
TOF transfer time [µs]	3	
MS^2 acquisition mode	dd-PASEF	
Total cycle time [s]	0.53	
Number of PASEF ramps	2 (cycle time 0.53 s)	
Target intensity	4000	
Active exclusion release [min]	0.1	
Collision Energy [eV]	20 & 50	

Table S5: Parameters of a comparable method on the TimsTof Pro 2 with ion mobility switched off (TimsOFF).

**Parameter**	**Positive mode method**	**Negative mode method**
Scan range (m/z)	75-1250	
Spectra rate [Hz]	4.00 Hz	
Deflection 1 Delta [V]	60	-60
Funnel 1RF [Vpp]	250	
Funnel 2RF [Vpp]	200	
Multipole RF [Vpp]	200	
Collision Energy (Collision Cell) [eV]	10	
Collision RF [Vpp]	450	
Quadrupole [eV], low mass (m/z)- MS^1	5 eV, 60 m/z	
TOF transfer time [µs]	65	
TOF transfer time [µs]	3	
Detection	Focus Mode	
MS/MS spectra rate control, limits	Dynamic (12-20 Hz)	
Target intensity (MS/MS TIC)	2000	
Collision Energy [eV]	20 & 50	
Total cycle time [s]	0.75	
Active exclusion	After 3 Spectra, release after 1 min	

Table S6: Parameters applied in Bruker TASQ software for annotation (ESI+ and ESI-).

Parameter	value
Determination ion quality	Principal ion only
Determination filter	Most mandatories and closest to RT
Mobilogram filter	Closest CCS value
Mobilogram ion quality	Principal ion only
Peak finder mode	Classic
Max/Min A/H upper limit	5
Min points over peak	4
Rt tolerance [min]	0.25
CCS tolerance [%]	7
Peak area threshold	100
EIC width [ppm]	5
Mobility calibration	With a list tuning mix ESI-TOF CCS compendium, intensity threshold 100, m/z range 0.05 Da
Mass calibration	With a list of sodium formate clusters, intensity threshold 1000, m/z range 0.05 Da

Table S7: Parameters applied in Bruker Metaboscape for feature list generation and annotation.

Parameter	ESI+	ESI-
Method of peak picking	T-ReX 4D Default Processing – Version 1.6	
Intensity threshold	1000	
Targeted Extraction Min Cluster Size	50	
Min Seed Cluster size	100	
EIC correlation for ion deconvolution	0.75	
Primary ion	[M+H]+	[M-H]-
Common ions	[M+H-H2O]+	[M-H-H2O]-
Seed ions	[M+Na]+, [M+K]+, [M+NH4]+	[M+Cl]-
RT Alignment Delta	10	
m/z alignment Delta	0.015	
Mobility alignment Delta	0.05	
RT grouping Delta	10	
m/z grouping Delta	0.015	
Mobilty grouping Delta	0.05	
MS/MS extraction method	Maxsum, group by collision energy	
Ion deconvolution, EIC correlation	0.75, primary ion [M+H]+	0.75, Primary ion [M-H]-

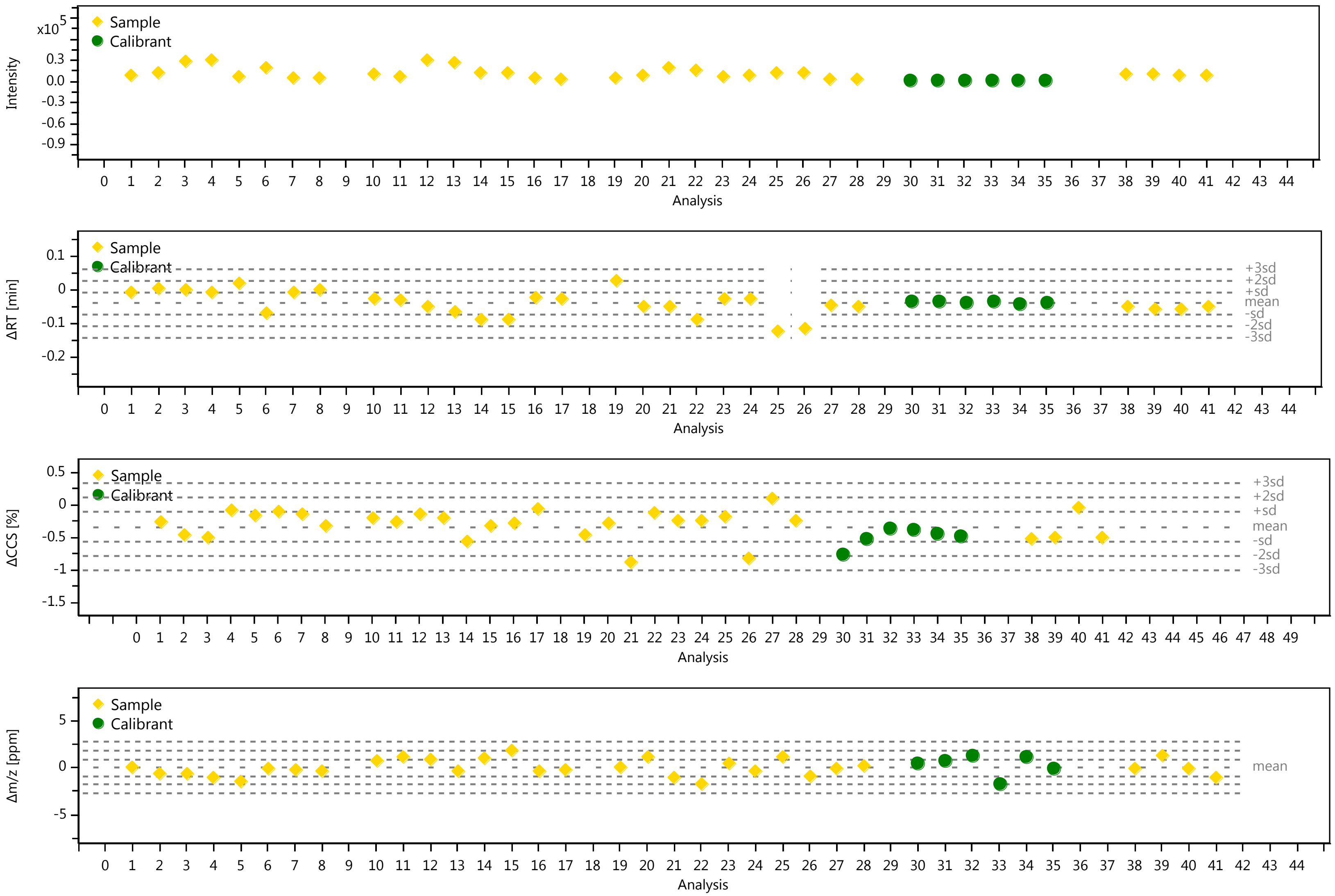

Figure S1: Overview of the m/z deviation, rt shift, and CCS error over the sample sequence for the internal standard Mono-isobuthylphthalate-D4 in negative ionization mode.

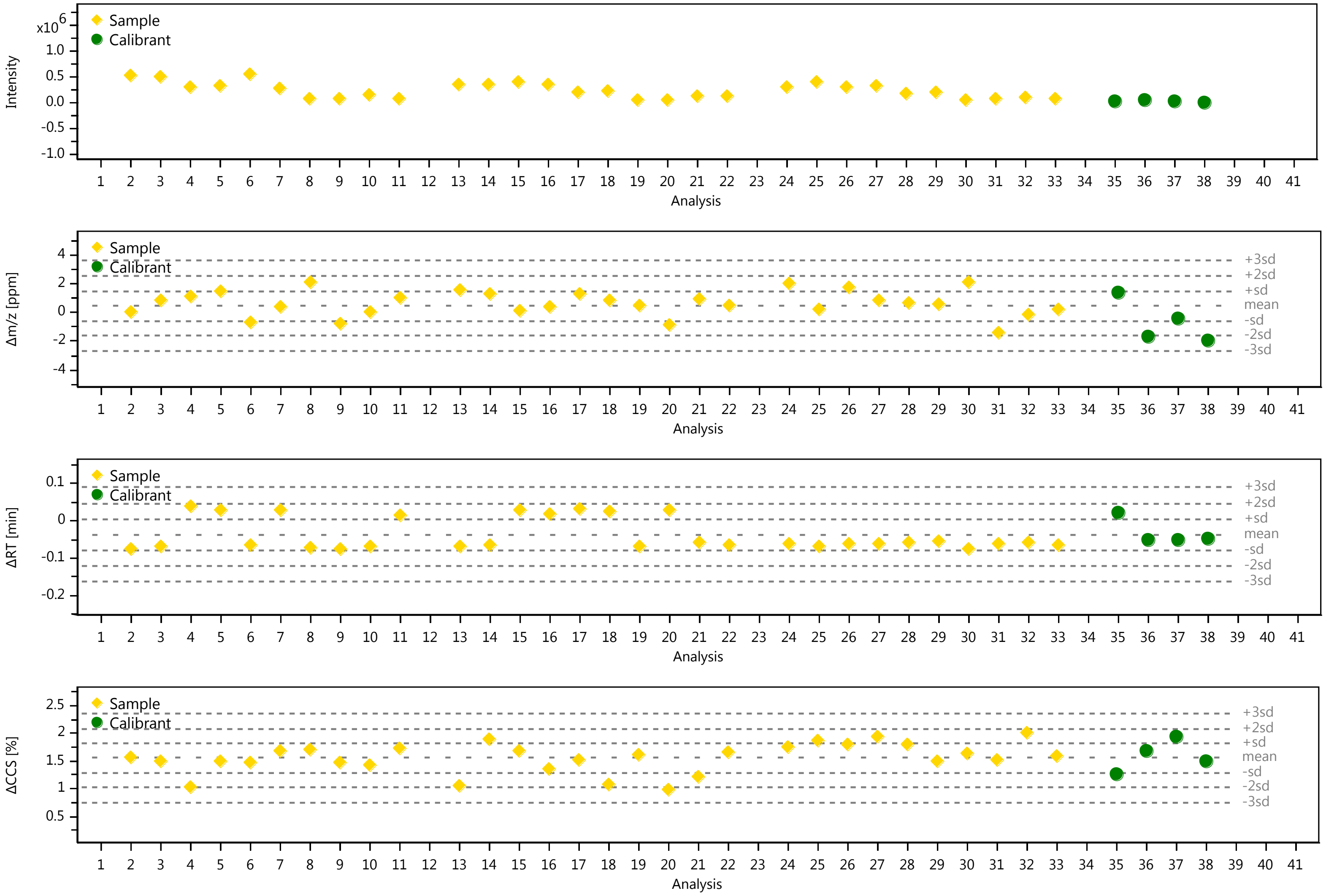

Figure S2: Overview of the m/z deviation, rt shift, and CCS error over the sample sequence for the internal standard Bezafibrate-D4 in positive ionization mode.

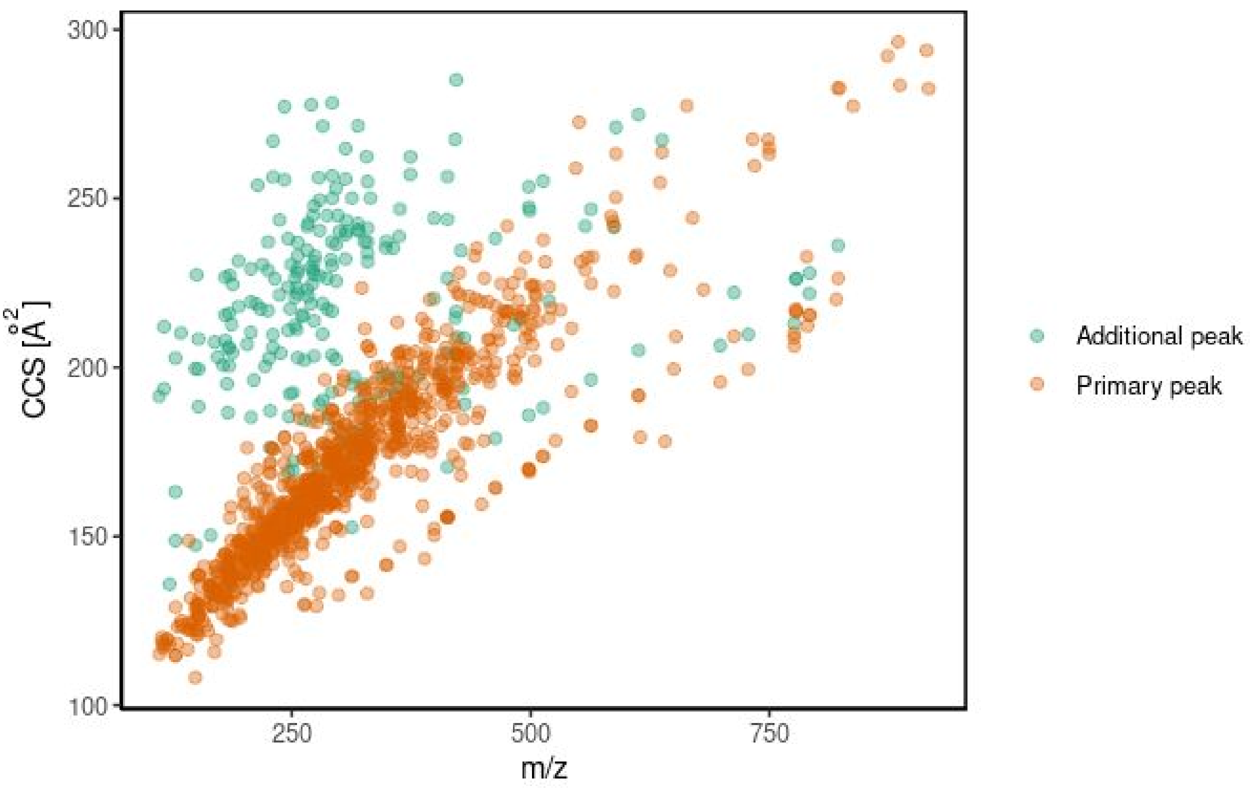

Figure S3: Additional peaks and selected peaks, most likely related to the monomer of each compound for the reference mixture (combined for $[M]^+$, $[M+H]^+$, and $[M-H]^-$).

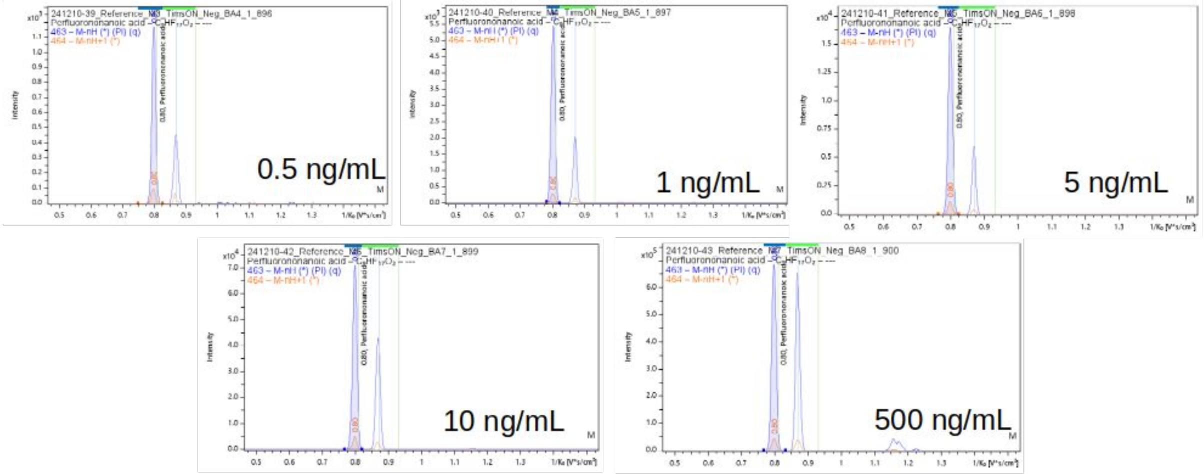

Figure S4: EIM of perfluorononaoic acid in ESI+ at five different concentrations as an exemplary compound showing several peaks in the EIM. Here, two peaks can be observed.

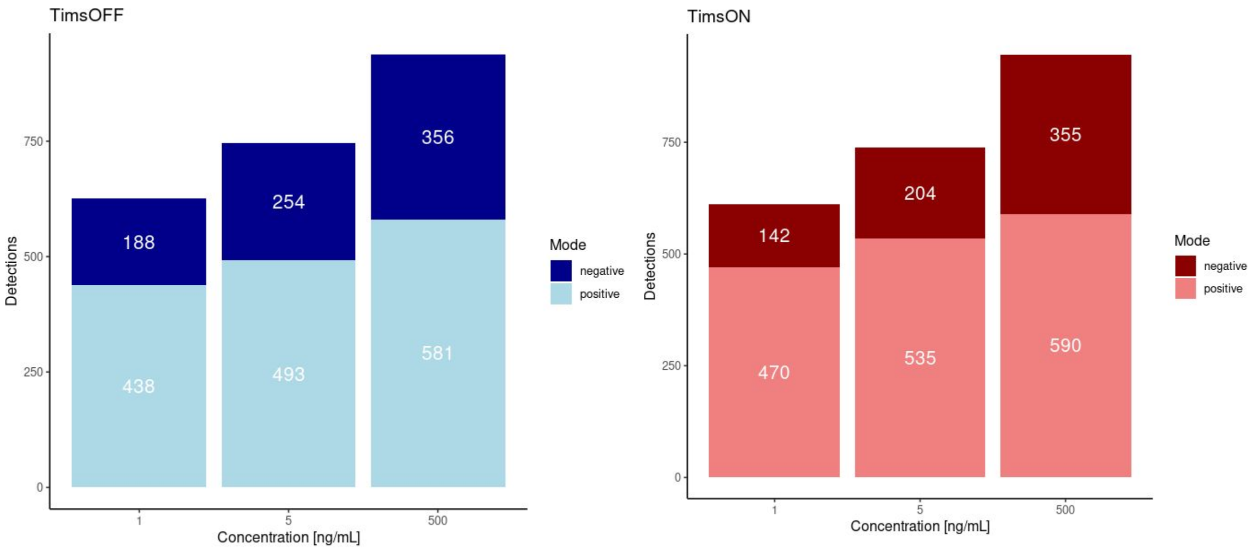

Figure S5: Number of detections (based on manual integration performed with the TasQ software) of the reference mixture measured in three different concentrations, measured with TimsON and TimsOFF, employing positive and negative ionization with a VIP-HESI source.

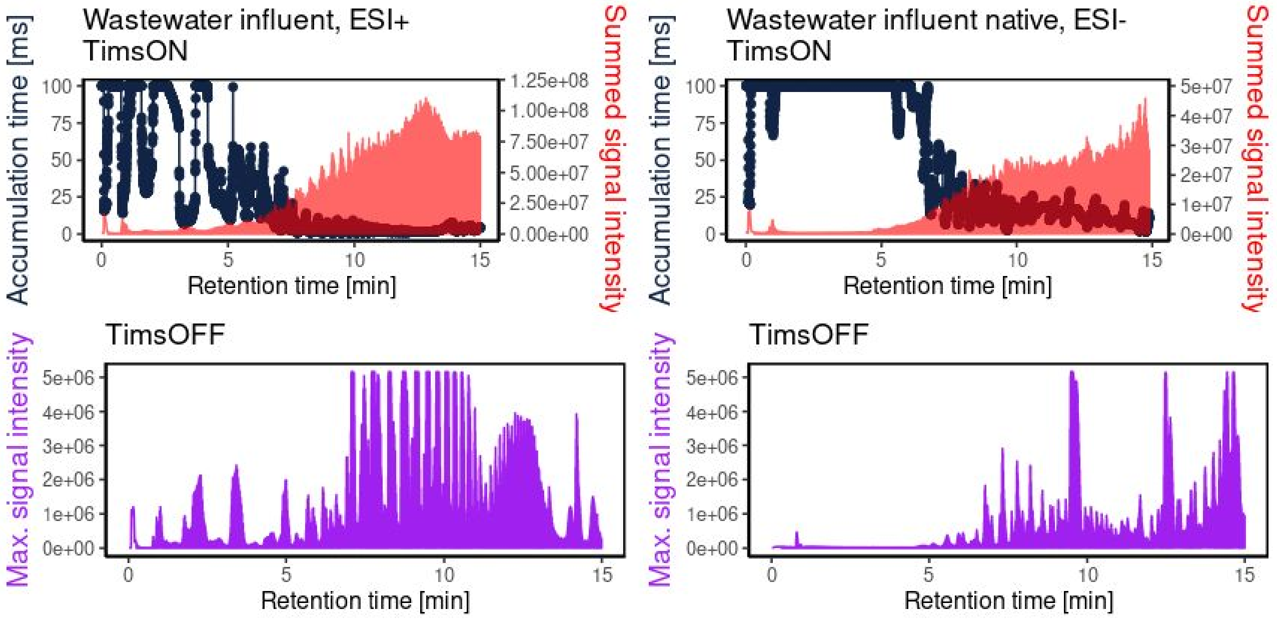

Figure S6: One exemplary example of a comparison between TimsOFF and TimsON with the same measurement settings and sample injection volume. The oversaturation of the detector at TimsOFF mode (see bottom) is avoided in TimsON mode by a decrease in the accumulation time (blue) based on the summed signal intensity (red).

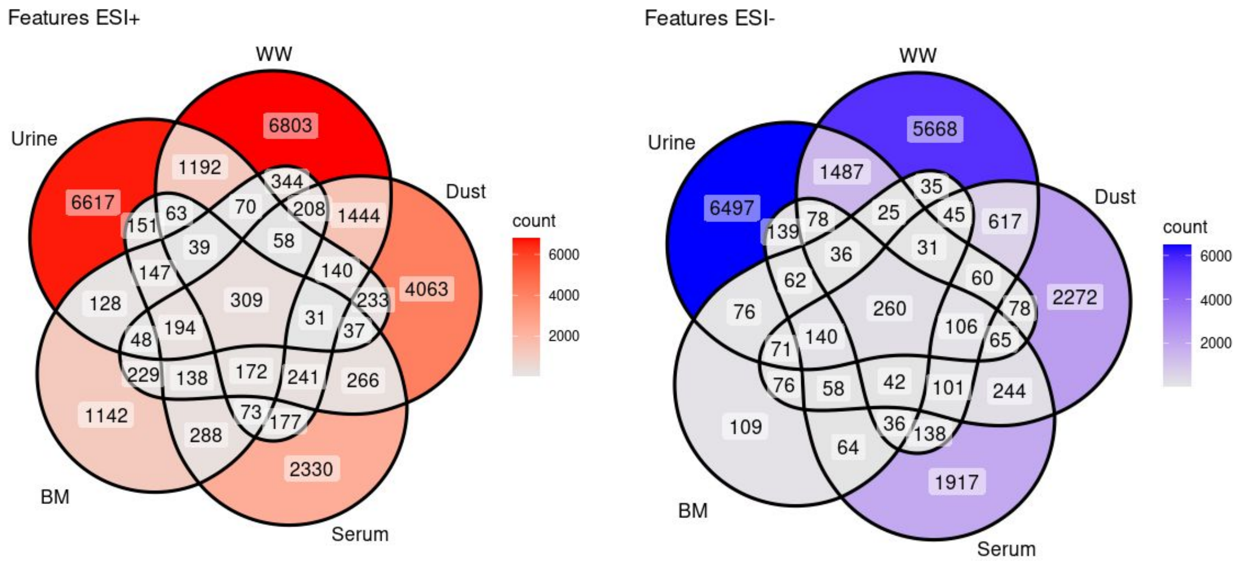

Figure S7: Characterization of the native
sample matrices as overlap of the non-target features after blank correction for positive and negative ionization mode (WW=wastewater influent, BM= breastmilk).

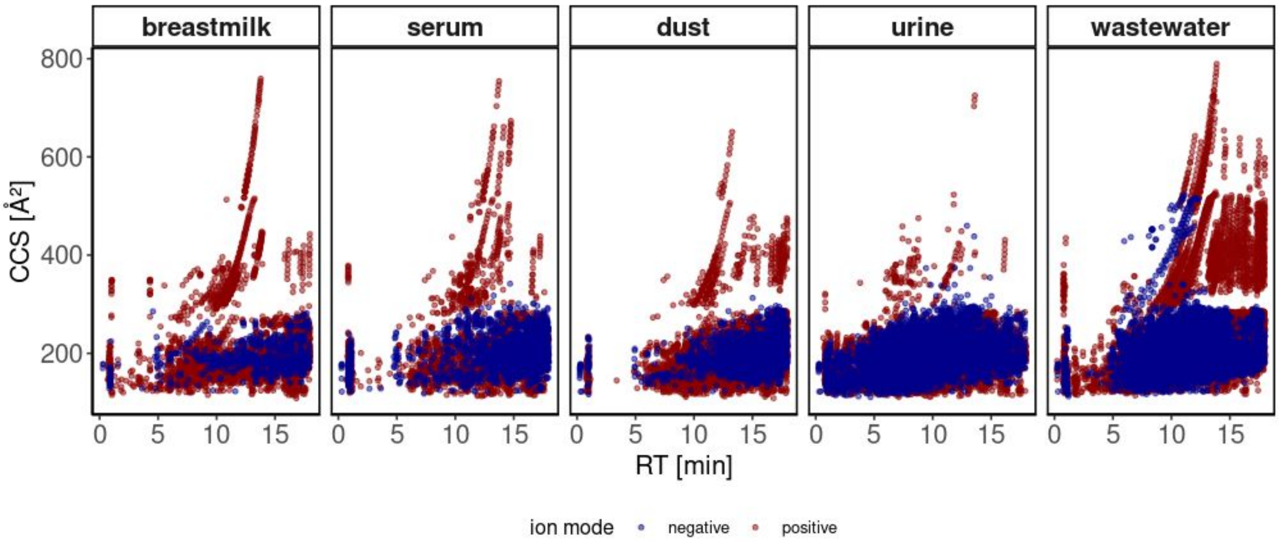

Figure S8: Retention time against CCS for five different sample matrices of the non-target feature list revealed in Metaboscape after blank correction. For single charged ions with CCS< 260 Å^2 no correlation to RT is observed, which reveals a high orthogonality between the two different dimensions of separation.

Table S8: Overview of the numbers of annotations based on different scenarios with given thresholds. Numbers are provided for ESI+ and ESI- respectively. Further information on the given numbers can be found in Tables SX3, SX4, and SX5.

	m/z match [ppm]	RT match [min]	CCS match [%]	MS^2 confirmation	Breast-milk	Serum	Dust	Urine	Waste-water	Blank
Confirmed annotation without interference (Schymanski level 1)	<5	±0.25	<3	yes	26/9	22/9	44/21	27/12	68/45	15/4
Annotation without MS^2 spectra (Schymanski level 4)	<5	±0.25	<3	no	24/10	11/7	17/22	19/8	29/17	0/0
Disturbing interference of annotation separated by CCS	<5	±0.25	<3 and interference > 5	no	6/4	10/0	23/2	18/3	6/3	0/0
False annotation differentiated by different CCS value	<5	±0.25	>5	no	6/0	9/0	3/1	5/5	5/1	0/0
False annotation in a suspect screening application (wider RT window)	<5	±2.5	>5	No	5/3	3/5	12/15	9/10	6/12	0/0

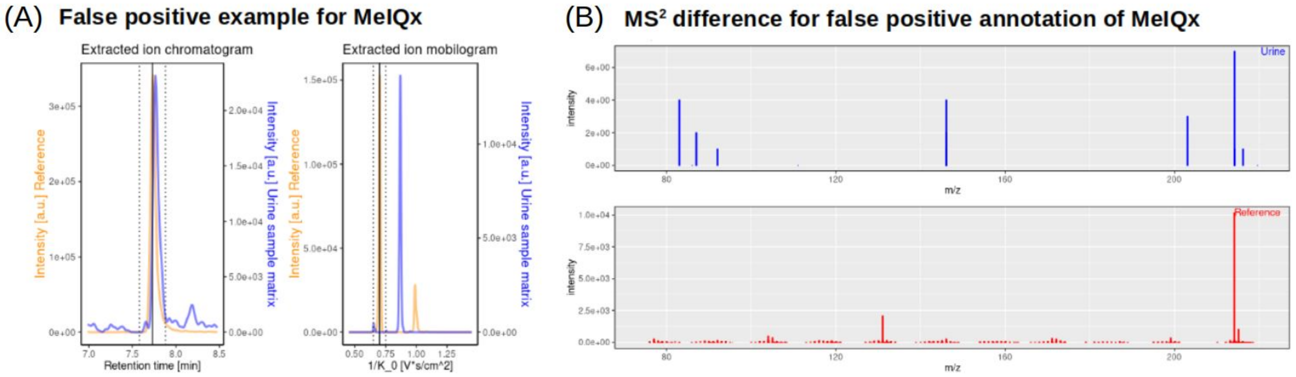

Figure S9: Example of the mutagenic and cancerogenic compound MeIQx, or 2-Amino-3,8-dimethylimidazo[4,5-f]quinoxalin. (A) Differences in EIC and EIM as already stated in the manuscript. (B) Difference in MS2 spectra extracted from the reference standard and the urine interference for two different ion mobility ranges, 0.8797-0.9338 V*s/cm^2 (blue) and 0.671-0.7195V*s/cm^2 (red).

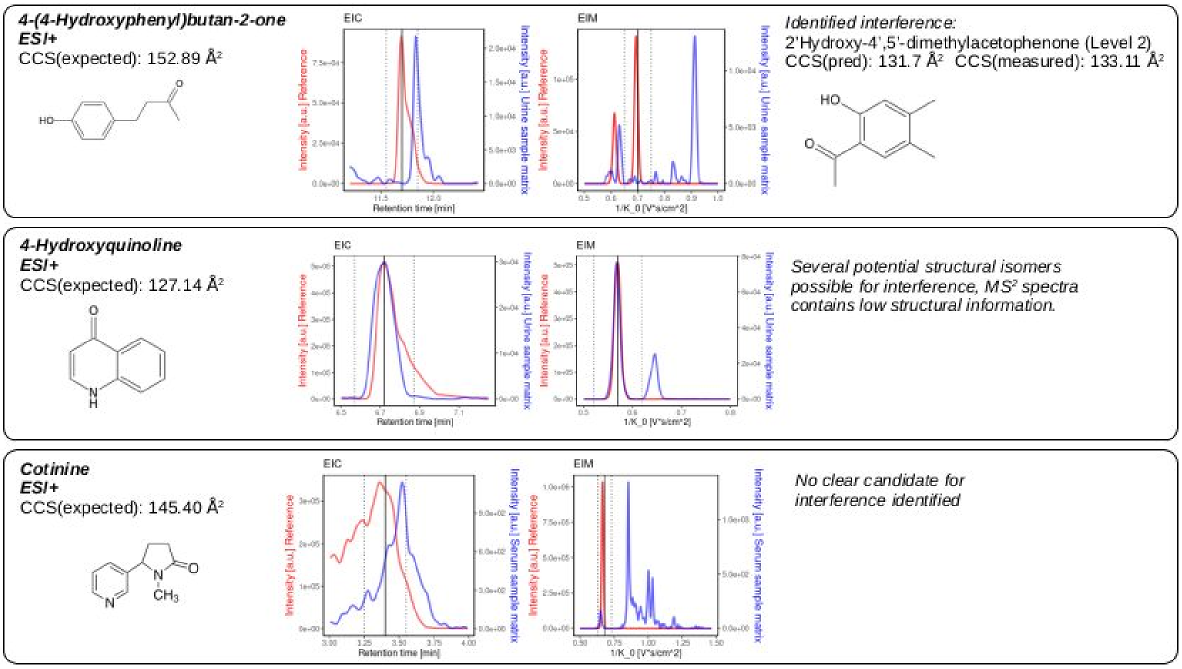

Figure S10: *Examples of analytes and interference that could be separated by TIMS and their structure elucidation.*

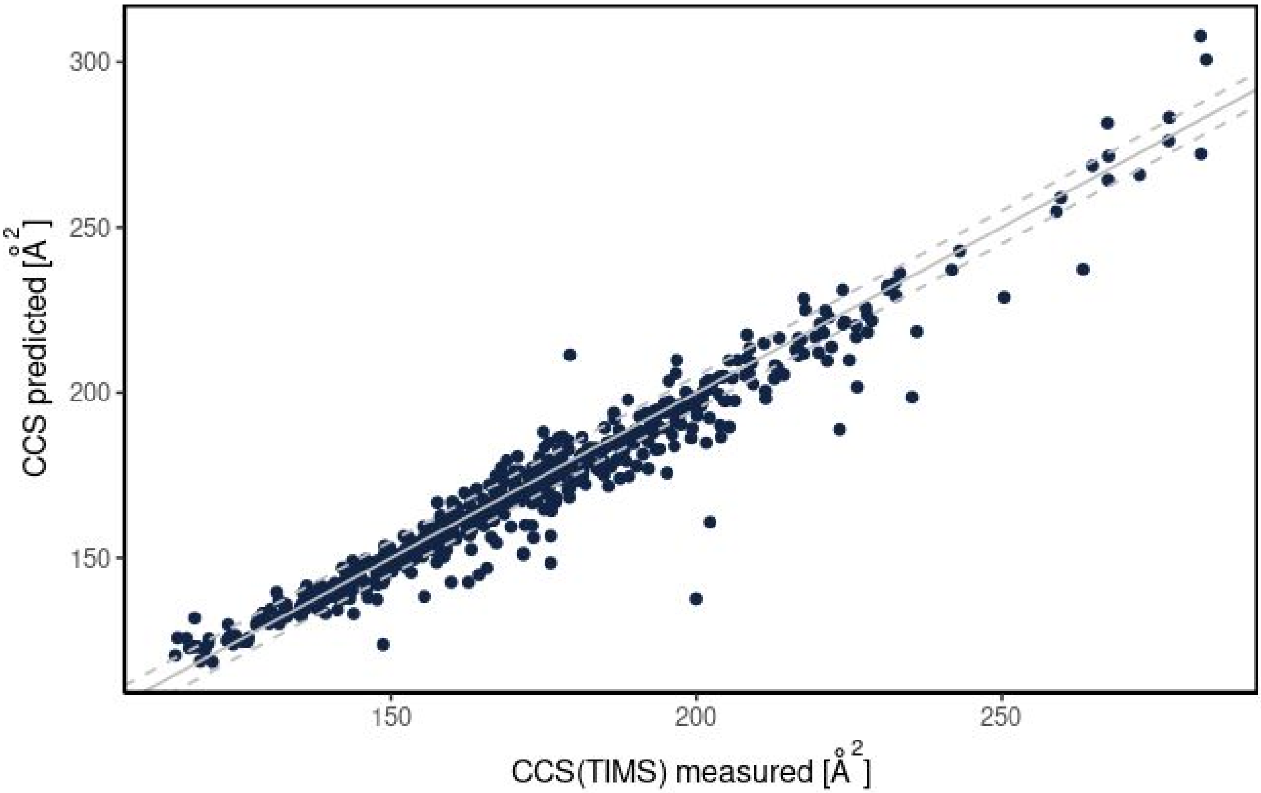

Figure S11: Experimental vs predicted CCS values (CCSbase) for all $[M+H]^+$ ions of the reference mixture. Grey dashed lines indicate the range ± the mean average error of ΔCCS= 4Å^2.

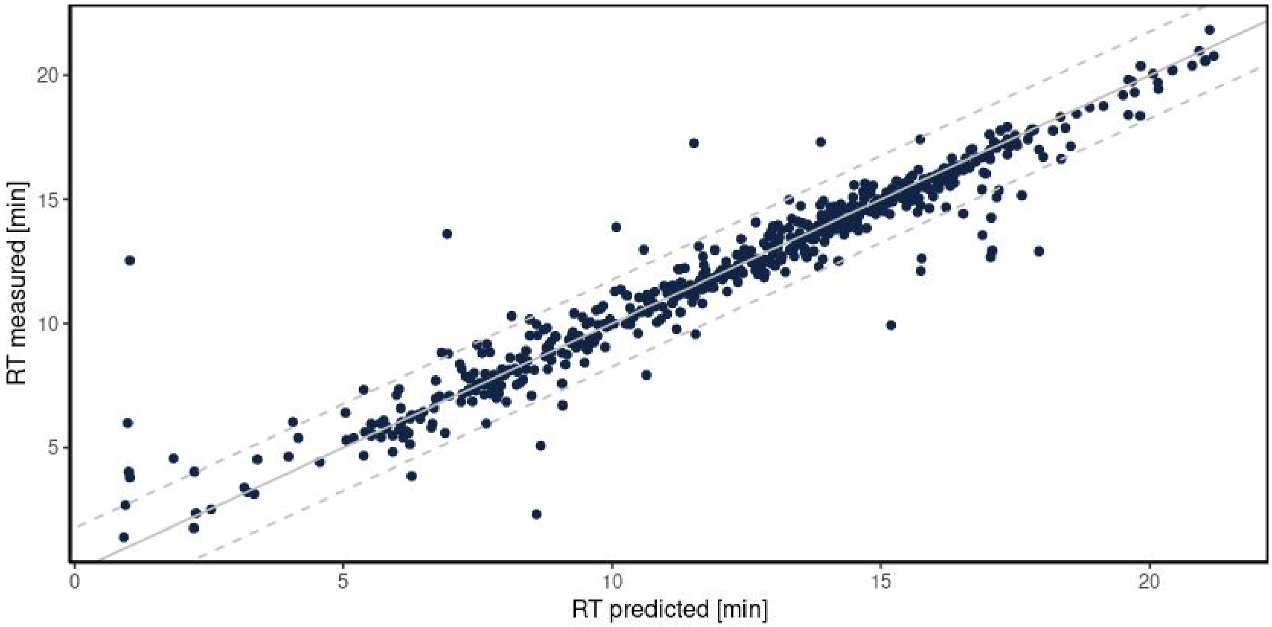

Figure S12: Experimental vs predicted RT values (RTPred) for all $[M+H]^+$ ions of the reference mixture. Note that the model does not report the split of test, training, and evaluation subsets. Therefore, the entire dataset (N = 595) is plotted. Grey dashed lines indicate the range ± the mean average error of ΔRT= 1.7 min.

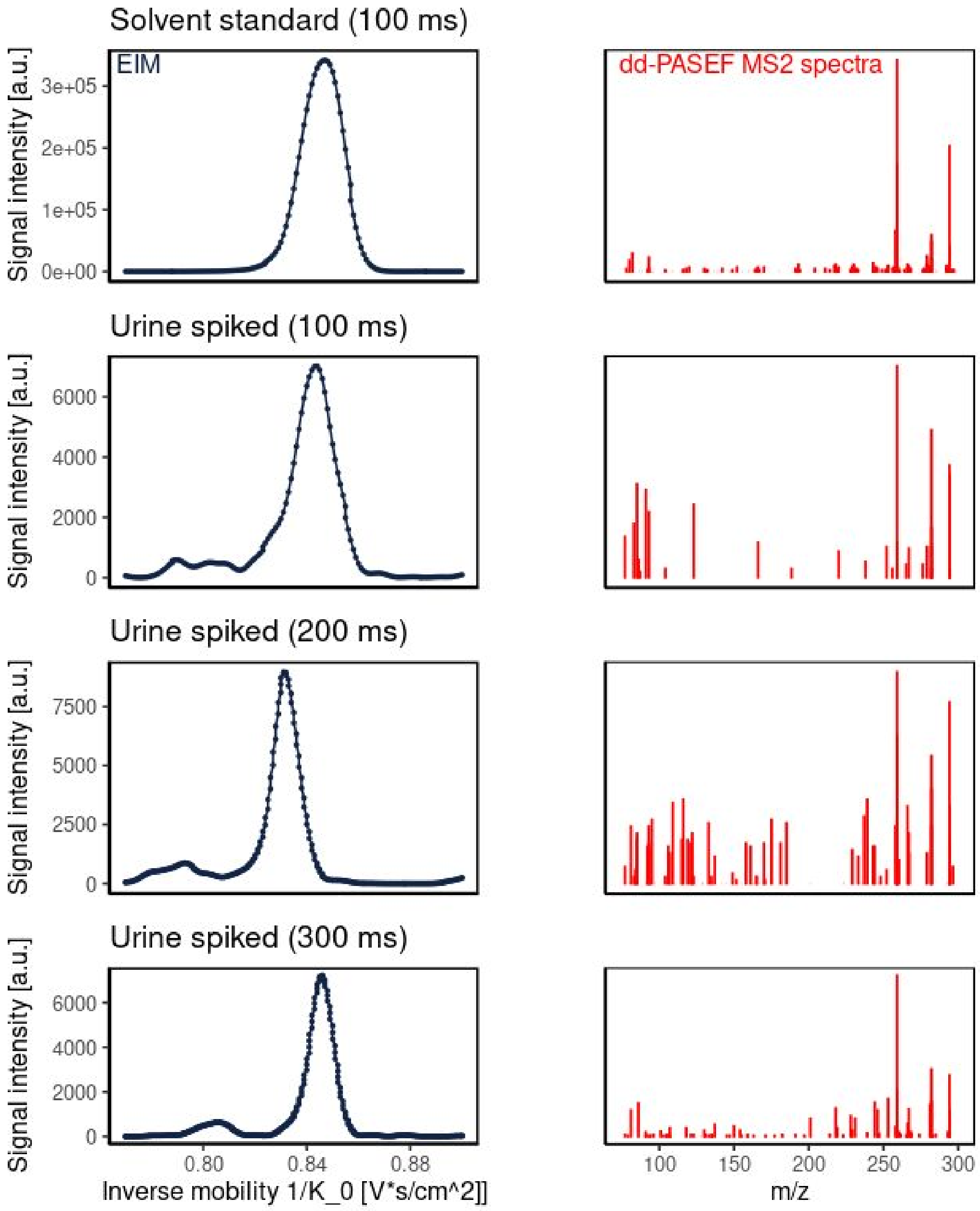

Figure S13: Extracted ion mobiligrams and acquired raw dd-PASEF MS2 spectra for desloratadine at three different ramp times for the high-level spiked urine sample compared at different ion mobility resolution (based on the applied ramp time for separation).

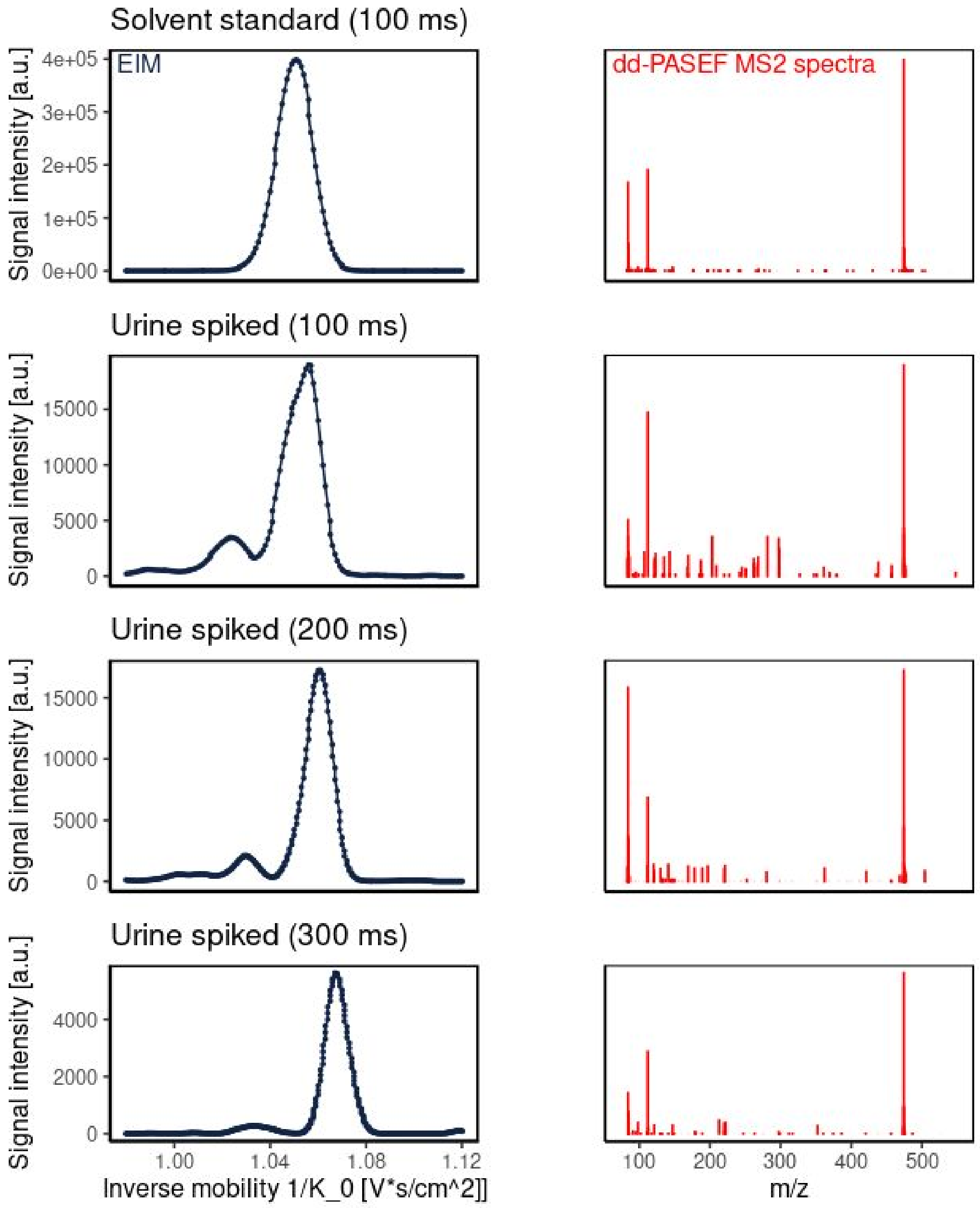

Figure S14: Extracted ion mobiligrams and acquired raw dd-PASEF MS2 spectra for raloxifene at three different ramp times for the high-level spiked urine sample compared at different ion mobility resolution (based on the applied ramp time for separation).
